# Mean corpuscular hemoglobin predicts the length of hospital stay independent of severity classification in patients with acute pancreatitis

**DOI:** 10.1515/med-2022-0559

**Published:** 2022-09-05

**Authors:** Hao Lin, Ting Yu, Rong Xu, Xing Li

**Affiliations:** Department of Clinical Science and Research, Zhongda Hospital, School of Medicine, Southeast University, Nanjing, 210009, China; Department of Gastroenterology, Zhongda Hospital, School of Medicine, Southeast University, Nanjing, China; Department of Quality Management, Zhongda Hospital, School of Medicine, Southeast University, Nanjing, China

**Keywords:** mean corpuscular hemoglobin, length of hospital stay, acute pancreatitis, patient management

## Abstract

Mean corpuscular hemoglobin (MCH) is a common blood routine test index. To explore the relationship between MCH and length of hospital stay in patients with acute pancreatitis (AP), we included 273 patients with AP without coronary heart disease, diabetes, hypertension and anemia in this study. All data were separated into three groups according to the length of hospital stay. Gender (*p* = 0.017) and severity classification (*p* < 0.001) were significantly correlated with length of hospital stay among three groups. Notably, MCH level was significant different among three groups (*p* = 0.009). Thus, all data were separated into two groups according to MCH level, and significant increases in the length of hospital stay were observed between two groups (*p* = 0.030). A positive correlation between length of hospital stay and MCH was observed (*r* = 0.172, *p* = 0.004). Multiple linear regression analysis showed that MCH was independent correlated with the length of hospital stay, no matter whether severity classification of AP was included (beta = 0.248, *p* < 0.001; beta = 0.212, *p* < 0.001). Our results demonstrated that the length of hospital stay was correlated with MCH level in patients with AP, and MCH level at admission may predict the length of hospital stay independent of severity classification in AP. These results may provide a potential evaluation basis for the management of patients with AP.

## 1 Introduction

Acute pancreatitis (AP) is an acute inflammatory disease of the pancreas, which can result in a systemic inflammatory response syndrome with significant morbidity and mortality, and the incidence rate of AP has increased in recent years [1]. AP is one of the most common gastrointestinal causes of hospitalization and results in family financial burden [2]. The prediction of hospitalization time of patients with AP is conducive to the management of patients and the optimization of clinical diagnosis and treatment efficiency. Noninvasive ultrasound examination is used in various diseases of the abdomen including AP [3]. However, there is a lack of simple indicators to predict the length of hospital stay of AP.

Severity classification is correlated with the length of hospital stay in patients with AP. Previous data showed that both Revised Atlanta Criteria and Determinant Based Criteria, which stratify AP by severity, strongly predict the length of hospital stay [4], but these indexes cannot predict the length of hospital stay at the beginning of hospitalization. A recent work carried out among children demonstrated that certain demographic and clinical factors, such as gender and ethnicity, were independently associated with the length of hospital stay for pediatric AP [5]. However, few laboratory parameters are available to evaluate the hospitalization in patients with AP.

Blood routine test is the most common clinical test at the beginning of hospitalization. Mean corpuscular hemoglobin (MCH) is the average amount of hemoglobin (HGB) per red blood cell and is calculated by dividing HGB by red blood cell (RBC) count. Mean corpuscular volume (MCV) is the average size of a red blood cell, and the mean corpuscular hemoglobin concentration (MCHC) is the average concentration of HGB per unit volume of red blood cells. The disease status of AP has a significant impact on blood routine test, and often abnormal hematological parameters predict adverse clinical events in patients with AP. For example, red cell distribution width (RDW) predicts the severity of patients with AP in pregnancy [6]. It has been reported that white blood cell (WBC) count could predict the severity of AP [7]. C-reactive protein predicts systemic inflammatory response syndrome and death in AP [8]. Total cholesterol concentration predicts the effect of plasmapheresis on hypertriglyceridemic AP [9]. Increased circulating total bile acid levels were associated with organ failure in patients with AP [10]. There are no studies investigating the efficacy of blood routine examination in predicting the length of hospital stay of AP. Here, the purpose of our study was to evaluate the relationship between hematological parameters and the length of hospital stay in patients with AP.

## 2 Materials and methods

### 2.1 Data collection

**Figure 1:**
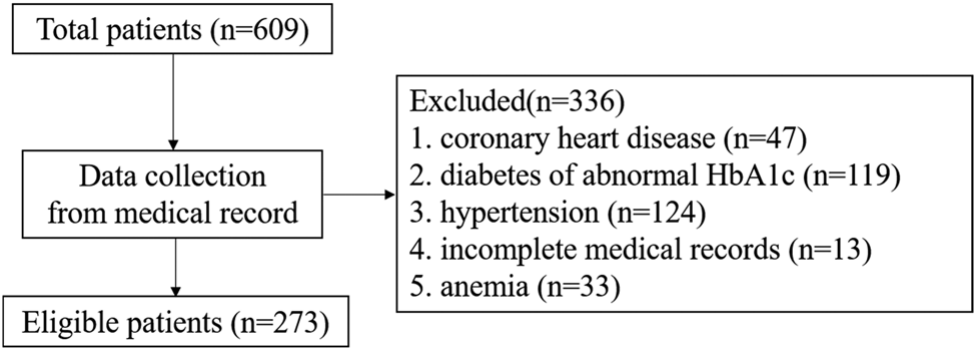
Details on the selection of patients.

Patients with AP from Zhongda Hospital, School of Medicine, Southeast University, were included in the retrospective study, and the diagnosis of all patients with AP was defined in accordance with the criteria of IAP/APA evidence-based guidelines [11]. Blood routine examination at admission were included, and the severity of disease was assessed by imaging test at admission. Patients with known coronary heart disease, diabetes and hypertension were excluded. Patients with anemia (HGB < 120 g/L for male and HGB < 110 g/L for female) were also excluded, and the details on the selection of patients are shown in Figure 1. Demographic and clinical characteristics of subjects were obtained from electronic medical records. Severity classification (mild, moderately, severely) was performed according to Marshall scoring system [12]. The study was approved by Ethics Committee for Clinical Research of Zhongda Hospital, Affiliated to Southeast University, and was performed according to the Declaration of Helsinki. The informed consent was waived by Ethics Committee for Clinical Research of Zhongda Hospital, Affiliated to Southeast University.

### 2.2 Statistical analysis

SPSS software version 23.0 was used to analyze all data. Continuous variables were shown as the mean ± standard deviation, and categorical variables were presented as percentages. The comparisons among three groups of continuous variables were performed by one-way analysis of variance. The comparisons of ordinal categorical variables were performed by rank sum test, and chi-square test was used to compare unordered categorical variables among three groups. Pearson or Spearman’s correlation analyses were employed to examine the correlations between the length of hospital stay and MCH appropriately. The multiple linear regression analysis was performed to analyze the independent effects of length of hospital stay in all subjects. A *p*-value of <0.05 was considered to be statistically significant.

## 3 Results

### 3.1 The clinical and laboratory features of patients with AP

Table 1 includes the characteristics of all participants. Because the average length of hospital stay for patients with AP was 14 days, and the average length of hospital stay in three-A class hospitals of Nanjing city was 8 days, we separated all data into three groups according to 8 and 14 days of length of hospital stay. Significant difference in the value distribution of gander (*p* = 0.017) and severity classification (*p* < 0.001) was found. We observed significant difference of MCH among three groups (30.02 ± 1.91, 30.21 ± 1.81, 30.92 ± 2.47 pg, *p* = 0.009). We also noticed significant difference of RBC among three groups (4.75 ± 0.45, 4.77 ± 0.56, 4.54 ± 0.62 × 10^12^/L, *p* = 0.007). There were no significant differences for other variables, including age, smoke, alcohol, cholecystectomy, WBC, HGB, RDW, MCV and MCHC.

**Table 1:** The clinical and laboratory data according to length of hospital stay

Length of hospital stay (days)	≤8	9–14	≥15	*p*-value
	*N* = 83	*N* = 95	*N* = 95	
Gender (male, %)	52, 62.7%	66, 69.5%	47, 49.5%	0.017*
Age (years)	44.0 ± 16.5	44.7 ± 16.1	46.7 ± 16.9	0.518
Smoke (*n*, %)	13, 15.7%	18, 18.9%	17, 17.9%	0.844
Alcohol (*n*, %)	11, 13.3%	16, 16.8%	13, 13.7%	0.754
Cholecystectomy (*n*, %)	3, 3.6%	6, 6.3%	6, 6.3%	0.666
Severity classification				<0.001***
Mild (*n*, %)	78, 28.6%	87, 31.9%	80, 29.3%	
Moderately (*n*, %)	1, 0.4%	1, 0.4%	0, 0%	
Severely (*n*, %)	4, 1.5%	7, 2.6%	15, 5.5%	
WBC (10^9^/L)	10.49 ± 5.36	11.01 ± 4.64	11.27 ± 4.58	0.559
RBC (10^12^/L)	4.75 ± 0.45	4.77 ± 0.56	4.54 ± 0.62	0.007**
HGB (g/L)	142.49 ± 14.44	143.85 ± 15.97	139.34 ± 17.38	0.141
RDW(%)	12.85 ± 0.92	13.01 ± 1.04	13.06 ± 0.87	0.310
MCV (fL)	88.89 ± 5.25	89.16 ± 4.93	90.18 ± 5.06	0.197
MCH (pg)	30.02 ± 1.91	30.21 ± 1.81	30.92 ± 2.47	0.009**
MCHC (g/L)	337.41 ± 10.86	338.93 ± 12.28	342.97 ± 21.84	0.054

The demographic characteristics and laboratory parameters of study participants (*n* = 273) according to tertiles of length of hospital stay (days). *p < 0.05; **p < 0.01; ***p < 0.001.

Due to the above interesting results between length of hospital stay and MCH, we separated all data into two groups according to the median number of MCH (Table 2). We observed significant increases in the length of hospital stay between two groups (11.95 ± 7.03, 13.96 ± 8.18 days, *p* = 0.030). We also noticed significant differences in gender (*p* = 0.029), RBC (*p* < 0.001), RDW (*p* < 0.001), MCV (*p* < 0.001) and MCHC (*p* < 0.001) between two groups. There were no significant differences for other variables, including age, smoke, alcohol, cholecystectomy, WBC, HGB and severity classification.

**Table 2:** The clinical and laboratory data according to MCH

MCH (pg)	≤30.3	＞30.3	*p*-value
	*N* = 142	*N* = 131	
Gender (male, %)	77, 54.2%	88, 67.2%	0.029*
Age (years)	44.3 ± 17.2	46.1 ± 15.7	0.363
Smoke (*n*, %)	21, 14.8%	27, 20.6%	0.207
Alcohol (*n*, %)	18, 12.7%	22, 16.8%	0.336
Cholecystectomy (*n*, %)	10, 7.0%	5, 3.8%	0.243
Severity classification			0.067
Mild (*n*, %)	132, 48.4%	113, 41.4%	
Moderately (*n*, %)	1, 0.4%	1, 0.4%	
Severely (*n*, %)	9, 3.3%	17, 6.2%	
WBC (10^9^/L)	10.65 ± 4.90	11.26 ± 4.78	0.294
RBC (10^12^/L)	4.83 ± 0.57	4.53 ± 0.50	<0.001***
HGB (g/L)	140.20 ± 16.57	143.68 ± 15.43	0.074
RDW(%)	13.19 ± 1.05	12.75 ± 0.77	<0.001***
MCV (fL)	86.60 ± 4.10	92.51 ± 4.19	<0.001***
MCH (pg)	29.04 ± 1.38	31.88 ± 1.76	<0.001***
MCHC (g/L)	335.36 ± 10.84	344.76 ± 19.14	<0.001***
Length of hospital stay (days)	11.95 ± 7.03	13.96 ± 8.18	0.030*

The demographic characteristics and laboratory parameters of study participants (*n* = 273) according to the median number of MCH. *p < 0.05; ***p < 0.001.

### 3.2 The correlation between length of hospital stay and laboratory data

**Figure 2:**
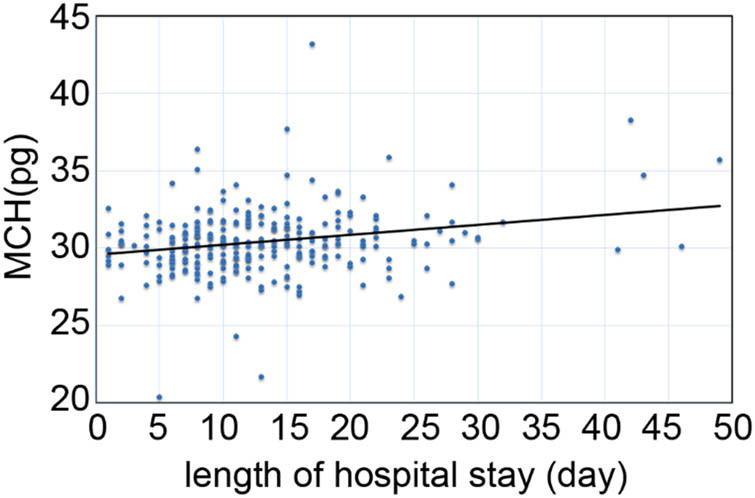
The correlation between length of hospital stay (length of hospital stay) and MCH of study participants (*n* = 273).

As shown in Figure 2, the positive correlation between the length of hospital stay and MCH was observed in all patients with AP (*r* = 0.172, *p* = 0.004). To further explore the correlation between length of hospital stay and other laboratory data, we did the correlation between the length of hospital stay and other laboratory data in whole population (Table 3). Length of hospital stay correlated with gender (*r* = −0.123, *p* = 0.042), severity classification (*r* = 0.182, *p* = 0.003), WBC (*r* = 0.128, *p* = 0.034) and RBC (*r* = −0.190, *p* = 0.002). Length of hospital stay had no correlation with age, smoke, alcohol, cholecystectomy, HGB, RDW, MCV and MCHC.

**Table 3:** The correlation between length of hospital stay and other laboratory data

	Correlation coefficient	*p*-value
Gender (male, %)	−0.123	0.042*
Age (years)	0.095	0.117
Smoke (*n*, %)	0.037	0.539
Alcohol (*n*, %)	0.018	0.763
Cholecystectomy (*n*, %)	0.044	0.467
Severity classification	0.182	0.003**
WBC (10^9^/L)	0.128	0.034*
RBC (10^12^/L)	−0.190	0.002**
HGB (g/L)	−0.088	0.146
RDW (%)	0.099	0.103
MCV (fL)	0.101	0.095
MCHC (g/L)	0.101	0.096
MCH (pg)	0.172	0.004**

*p < 0.05; **p < 0.01.

### 3.3 The correlation analyses in multiple linear regression analysis

The correlation analyses were carried out in all subjects. The basic demographic characteristics, such as gender, age and AP severity, may influence the length of hospital stay [5]. The laboratory parameters of study participants, such as WBC, HGB and RDW, might be associated with the length of hospital stay as well. Therefore, gender, age, severity classification, WBC, HGB, RDW and MCH were included as independent variables in multiple linear regression analysis. We did two regression models to investigate the correlation between length of hospital stay and laboratory data (Table 4). Gender, age, WBC, HGB, RDW and MCH were adjusted in model 1, and model 2 added severity classification on the basis of model 1. The results found that length of hospital stay was correlated with MCH in both model 1 and model 2 (beta = 0.248, *p* < 0.001; beta = 0.212, *p* < 0.001). Length of hospital stay was independently correlated with severity classification and gender (beta = 0.212, *p* < 0.001; beta = 0.211, *p* = 0.001; beta = −0.171, *p* = 0.015), as shown in Table 4.

**Table 4:** The factors related to length of hospital stay in patients with AP in multivariable linear regression analysis

	Unstandardized coefficients		Standard beta	*p*-value
	*B*	SE		
**Model 1**				
Gender	−3.608	1.087	−0.231	0.001**
Age	0.024	0.028	0.052	0.382
WBC	0.225	0.093	0.143	0.016*
HGB	0.026	0.035	0.056	0.444
RDW	0.898	0.495	0.111	0.071
MCH	1.016	0.224	0.281	<0.001***
**Model 2**				
Gender	−2.854	1.091	−0.183	0.009*
Age	0.017	0.027	0.036	0.542
WBC	0.142	0.094	0.090	0.134
HGB	0.005	0.035	0.011	0.882
RDW	0.783	0.488	0.097	0.109
Severity classification	2.639	0.795	0.204	0.001**
MCH	0.874	0.224	0.242	<0.001***

Model 1: linear regression based on gender, age, WBC, HGB and MCH.

Model 2: linear regression based on gender, age, WBC, severity classification, HGB and MCH.

*p < 0.05; **p < 0.01; ***p < 0.001.

## 4 Discussion

We found that length of hospital stay had a relationship with MCH level in patients with AP, and the multiple linear regression analysis showed that MCH level positively correlated with the length of hospital stay independent of severity classification of AP. It has been reported that a few of biomarkers are associated with the length of hospital stay in patients with AP. Serum lactate dehydrogenase (LDH) and its isozyme hydroxybutyrate dehydrogenase (HBDH) were early predictive markers of the severity of AP [13,14]. LDH and its isozyme HBDH show more affinity for α-ketoacid and associated with severity of AP [14,15]. Specific inflammatory cytokines, such as IL-6 and TNF-α, are used to detect inflammation in many cystic conditions and can be used in pancreatitis as well as to distinguish tumors from inflammation in all cystic formations [16,17,18]. IL-6 and TNF-α damaged hematopoietic cells through necrosis as well as other toxic products, leading more LDH and HBDH release [17,18]. Because the markers above have been proved to be related to the severity of AP, thus, these markers are likely to predict the length of hospital stay.

The mechanisms of MCH as a predictor of length of hospital stay in AP are not fully clear. However, some hypotheses can be proposed. One potential explanation is that our study did not exclude patients with liver and kidney dysfunction, which caused a decrease in erythropoietin levels [19]. The body may tend to produce more and smaller red blood cells, while the HGB remains unchanged [20]. And this phenomenon may not be related to inflammatory reaction [21].

Indeed, the severity classification of AP was a strong risk factor of length of hospital stay in patients with AP, and the more serious the patient was, the longer they stayed in hospital (Table 1). The length of hospital stay is certainly associated with complications of pancreatitis, toxic spills from nectotic changes and inflammation, cystic hemorrhagic bleeding and secondary inflammation that occurs in a large number of cases [22,23]. All these factors above are related to the severity of AP. Therefore, our study considered the severity classification of AP as the adjustment variable in multiple linear regression analysis. Our analysis showed that the length of hospital stay was related to MCH regardless of severity classification in regression equation. We also found that gender was another risk factor of length of hospital stay. The female patients stayed longer than male ones (Table 1), and this phenomenon consisted with previous report in patients with pediatric AP [5]. Besides, it was reported that RDW was an independent risk factor for AP associated lung injury (APALI) [24]. We noted a significant difference for RDW between two groups divided by MCH in Table 2 (*p* < 0.001), but there is no significant difference for RDW among three groups divided by the length of hospital stay in Table 1. Based on above findings, after adjustment for severity classification, gender and RDW, length of hospital stay still positively correlated with MCH (*p* < 0.001, Table 4). Therefore, our study confirmed that MCH is a risk factor independent of severity classification, gender and RDW in AP.

To exclude the effects of anemia on the correlation between MCH and length of hospital stay in patients with AP, patients with AP with anemia were excluded in this study. Interesting, after excluding patients with AP with anemia, the statistically correlation between length of hospital stay and MCH still was observed in patients with AP, and HGB was not the risk factor of length of hospital stay according to the multiple linear regression analysis (Table 4).

The present results had some limitations. First, serum samples of patients could not be obtained, so that we could not measure serum concentration of inflammation markers, such as IL-6 and TNF-α. Second, considering different medical treatment of different hospitals, there were differences among the treatment of AP. Although most patients will be treated according to clinical guidelines in different medical institutions, it is still possible to affect the relationship between MCH and length of hospital stay in our study. Third, the patients’ economic position and treatment willingness may be a potential confounding factor that affects our final outcomes in patients with AP.

In conclusion, the evaluation of length of hospital stay is conducive to the management of patients by hospital. We demonstrate that length of hospital stay is correlated with the MCH level independent of severity classification in patients with AP, and the MCH level at admission may have an effect on predicting the length of hospital stay in patients with AP.
